# Purple Urine Bag Syndrome: A Rare and Surprising Clinical Presentation

**DOI:** 10.7759/cureus.33354

**Published:** 2023-01-04

**Authors:** Nathan DeRon, Carley Legan

**Affiliations:** 1 Internal Medicine, Methodist Health System, Dallas, USA

**Keywords:** purple, urinary tract infection, uti, purple urine bag syndrome, pubs

## Abstract

Purple urine bag syndrome (PUBS) is a rare finding that can be very alarming to patients and physicians. PUBS has a simple visual diagnosis with clinical symptoms that can aid in a quick and appropriate treatment plan. However, a lack of physician awareness could be harmful to the patient and cause unnecessary treatment and increased morbidity and financial burden to the patient. We present a case with this surprising finding and discuss the pathophysiology and management options for this rare syndrome.

## Introduction

Purple urine bag syndrome (PUBS) is a rare clinical presentation that physicians may encounter only sparingly. It is a rare, but typically benign, clinical syndrome in which a patient’s urinary catheter bag exhibits purple urine [1]. It is the result of bacteriuria, whether asymptomatic or causing urinary tract infection (UTI) symptoms, which causes discoloration of the urine seen in a Foley bag. The pathophysiology involves an ongoing bacterial infection in which specific enzymes cause blue- and red-pigmented breakdown products that combine to form grossly purple urine [2]. Here, we present a rare case of PUBS in an elderly patient with a history of long-term catheterization and constipation.

## Case presentation

A 79-year-old female with a past medical history of urinary retention managed with a chronic indwelling Foley catheter, recurrent urinary tract infections (UTIs), hypertension, prior stroke with residual dysarthria, and dementia presented to the hospital due to altered mental status noticed at home by her family. The patient was confused but able to provide a limited history that was supplemented by the family who was present at the bedside.

The family noted a change in the patient’s urine color on the day prior to admission. Soon after the change in urine color, the patient’s family noticed the patient becoming increasingly confused. The patient expressed discomfort in her lower abdomen. The family reported that the patient lives at home with her family, is wheelchair-bound, and has a chronic indwelling Foley catheter due to a history of stroke that resulted in a neurogenic bladder.

**Figure 1:**
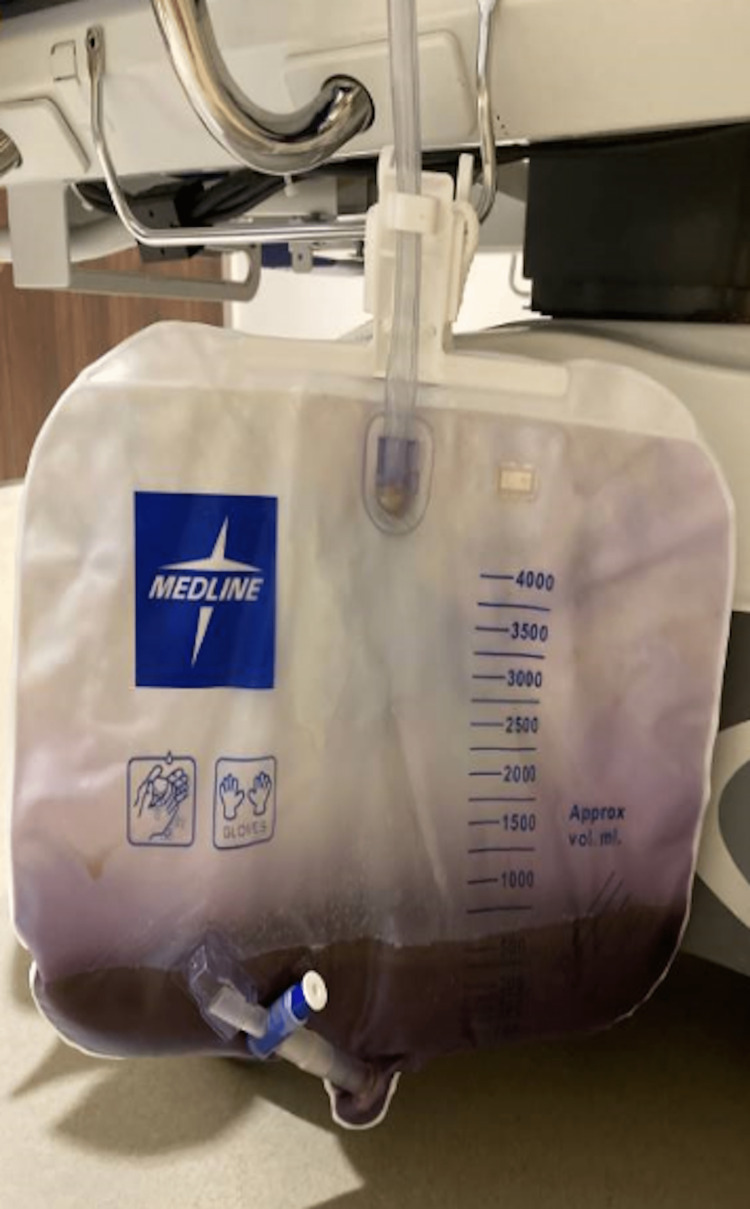
The patient’s Foley bag displaying purple urine, which was present on the patient’s arrival at the emergency department.

The patient’s vital signs were unremarkable. The physical examination was significant for a confused, thin, elderly female in no acute distress, dry mucous membranes, active bowel sounds, tenderness to deep palpation of the lower abdomen, left-sided upper and lower extremity weakness, and a Foley bag with purple urine as exhibited in Figure 1. The patient’s laboratory findings were significant for normocytic anemia with a hemoglobin level of 11 mg/dL. Urinalysis revealed cloudy urine that was positive for nitrites and leukocyte esterase and had greater than 100 white blood cells per high-power field. Computed tomography imaging of the abdomen and pelvis showed fluid-filled loops of the small bowel that were borderline dilated suggestive of a developing ileus or small bowel obstruction.

The patient was started on empiric antibiotics, including ceftriaxone, based on prior urine culture data. The Foley catheter was exchanged, and intravenous hydration and an aggressive bowel regimen were started. Within 24 hours of therapy, the patient had significant improvement in mentation. Within 48 hours, the patient returned to baseline mentation per the family. Her urine color returned to clear yellow, and she had numerous bowel movements and was safely tolerating oral nutrition intake.

The urine culture was positive for *Proteus mirabilis* and *Escherichia coli*. The patient completed an antibiotic treatment course for a complicated UTI according to culture sensitivities and was discharged with outpatient follow-up.

## Discussion

**Figure 2:**
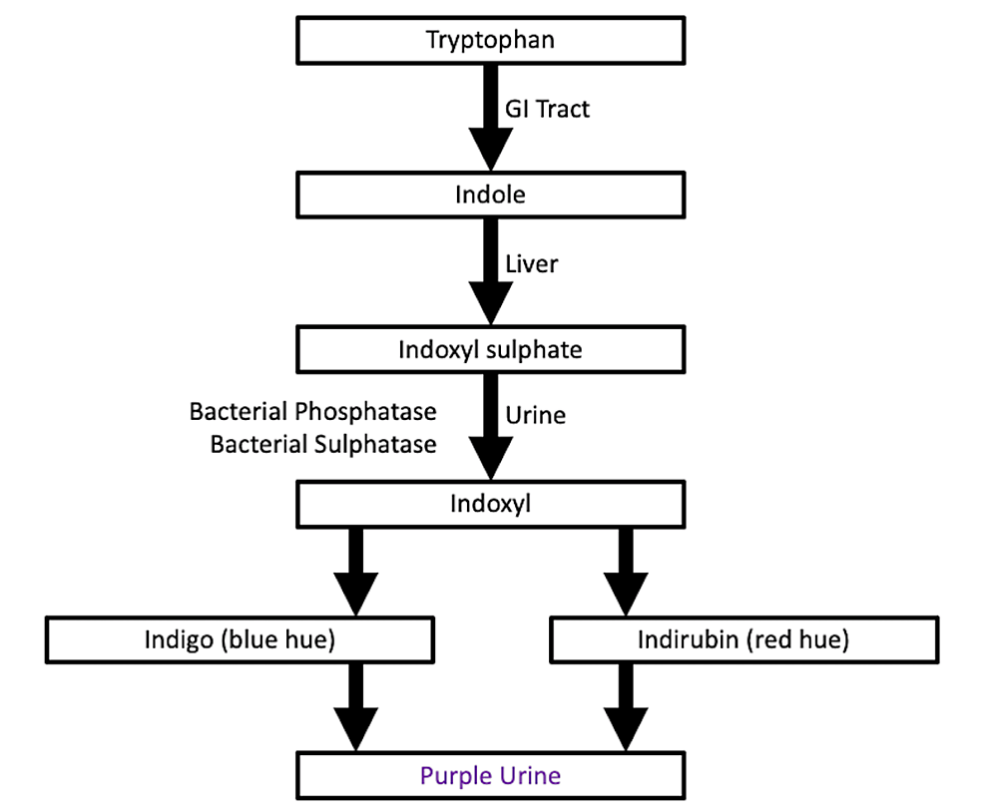
Flowchart illustrating the biochemical pathway resulting in purple urine bag syndrome. GI: gastrointestinal

PUBS is a mostly benign manifestation of bacterial colonization of the urinary tract in patients with chronic urinary catheterization [1]. The purple coloration is the result of a mixture of indirubin, which provides a red color, and indigo, which provides a blue color. As these two substances mix in the urine, the result is a purple hue [2]. Both indirubin and indigo are produced by tryptophan metabolism in the gastrointestinal tract as exhibited by the flowchart in Figure 2. Tryptophan is metabolized to indole, which is then metabolized to indoxyl sulphate in the liver. Indoxyl sulphate is metabolized to indoxyl in the urine with assistance from bacteria, which produce phosphatase and sulphatase. Indoxyl is subsequently transformed into indigo and indirubin in the setting of alkalinized urine and dehydration [3].

PUBS is associated with bacteria that produce an alkaline environment such as *Providencia*, *Klebsiella*, *Proteus*, and Enterobacteriaceae [4]. However, evidence of purple urine does not necessarily equate to a UTI. Purple urine may be evident upon bacterial colonization without infectious symptoms in asymptomatic bacteriuria. The presence of infectious symptoms is frequently difficult to elucidate as PUBS often occurs in elderly demented patients with chronic urinary catheters and risk factors such as immobility, constipation, and chronic kidney disease [5,6].

PUBS itself is not an indication for therapy. PUBS need only be treated if the patient has symptoms related to the bacteria found in the urine. The clinical course of PUBS is benign and typically resolves with acidification of the urine either through self-resolution or antibiotic therapy [7]. Antibiotic therapy in symptomatic individuals is targeted to the organisms growing on urine culture. Antibiotic susceptibility of the organisms is extremely helpful in these cases as the bacteria causing PUBS are often resistant to many antibiotics given the setting of chronic catheterization and the likelihood of recurrence and development of resistance [8].

Solutions to PUBS mostly rely on prevention. The prevention of catheter-associated UTIs includes shortening the duration of catheterization when possible. Also, eliminating blockages in urinary catheters reduces the risk of infection when urine is allowed to flow through the catheter tubing as designed [9]. Avoiding chronic indwelling catheters and employing periodic catheter exchanges are two of the most common recommendations to avoid UTIs and PUBS.

## Conclusions

PUBS is a rare and benign condition associated with bacterial colonization of chronic urinary indwelling catheters. The condition typically indicates the presence of bacterial overgrowth in the urinary tract. The bacterial burden may or may not cause symptoms and lead to infection. The preferred therapy for PUBS is observation unless the bacterial organism causes urinary symptoms in which urine analysis, urine culture, and antibiotic administration are indicated.
